# Training Inference Making Skills Using a Situation Model Approach Improves Reading Comprehension

**DOI:** 10.3389/fpsyg.2016.00116

**Published:** 2016-02-15

**Authors:** Lisanne T. Bos, Bjorn B. De Koning, Stephanie I. Wassenburg, Menno van der Schoot

**Affiliations:** Department of Pedagogical and Educational Sciences, Section of Educational Neuroscience, Faculty of Behavioral and Movement Sciences & LEARN! Institute, Vrije Universiteit AmsterdamAmsterdam, Netherlands

**Keywords:** reading comprehension, intervention studies, inference making, situation model, primary school children

## Abstract

This study aimed to enhance third and fourth graders’ text comprehension at the situation model level. Therefore, we tested a reading strategy training developed to target inference making skills, which are widely considered to be pivotal to situation model construction. The training was grounded in contemporary literature on situation model-based inference making and addressed the source (text-based versus knowledge-based), type (necessary versus unnecessary for (re-)establishing coherence), and depth of an inference (making single lexical inferences versus combining multiple lexical inferences), as well as the type of searching strategy (forward versus backward). Results indicated that, compared to a control group (*n* = 51), children who followed the experimental training (*n* = 67) improved their inference making skills supportive to situation model construction. Importantly, our training also resulted in increased levels of general reading comprehension and motivation. In sum, this study showed that a ‘level of text representation’-approach can provide a useful framework to teach inference making skills to third and fourth graders.

## Introduction

Generally, it is recognized that many primary school children fail to attain a sufficient reading comprehension level. The National Center for Education Statistics (2011), for example, reports that 33% of all fourth-grade children and 24% of all eighth-grade children in the United States perform below the required standards. Periodical analyses of children’s reading comprehension performance in the Netherlands have shown similar results (e.g., van der Schoot, 2008). One way to enhance reading comprehension is to teach children how they can use reading strategies (see Pressley, 2000, for a review on comprehension instruction). Over the years, various reading strategies have been suggested to improve text comprehension (Trabasso and Bouchard, 2002; van der Schoot et al., 2008; De Koning and van der Schoot, 2013). However, a gap remains between research findings and educational practice (Van Keer and Verhaeghe, 2005; Liang and Dole, 2006; Andreassen and Bråten, 2011). That is, instructional methods and materials to teach reading comprehension often are insufficient and the empirical support for the taught reading strategies’ effectiveness is equivocal (Droop et al., 2012; Houtveen and van de Grift, 2012; Stoeldraijer and Forrer, 2012). In particular, it has been suggested that instructional methods have not been sufficiently grounded in relevant reading comprehension literature focusing on the different levels of mental text representation. For example, van der Schoot et al. (2010) argued that there are astonishingly few studies directly examining the type of instructional support that encourages readers to construct a situation model from text (see Zwaan and Radvansky, 1998, for a review on situation models). To help fill this gap in the literature, the present study aimed to improve children’s text comprehension at the situation model level. To achieve this, we tested a reading strategy training which was developed to target inference making skills, which are widely considered to be the key factor in situation model construction (e.g., Kintsch and Rawson, 2005; van den Broek et al., 2011; McMaster et al., 2012; van den Broek and Espin, 2012). Noticeable, the present training was part of a broader large-scale reading comprehension intervention aimed at improving children’s ability to form a situation model of text. In this intervention, three crucial situation model strategies were trained: (1) inference making [focusing on (re-)establishing coherence in situation models], (2) comprehension monitoring (focusing on situation model updating), and (3) mental simulation [focusing on the (re-)enactment of perceptual- and motor experiences]. The effects of the latter two reading strategy training interventions are reported elsewhere (respectively, Wassenburg et al., 2015; De Koning et al., submitted). Here, we present the aim, content, and outcomes of the first one (i.e., inference making).

### Situation Model Theory and Inference Making

According to research on reading comprehension, a text can be represented at three levels: the surface representation, the textbase representation, and the situation model representation (Kintsch and van Dijk, 1978; Kintsch, 1988). The surface representation consists of the verbatim words and clauses extracted from the text. At the textbase level, the meanings of words and clauses are processed and subsequently stored in the reader’s memory. A situation model representation is a coherent and non-linguistic mental representation of the ‘state-of-affairs’ described in a text, rather than a mental representation of the text itself (Zwaan and Radvansky, 1998; van der Schoot et al., 2010; van den Broek et al., 2011). During text comprehension, readers construct a situation model representation by monitoring various basic narrative dimensions such as protagonist, time, space, causality, and intentionality (Zwaan et al., 1995; van der Schoot et al., 2012). Integrating information from these dimensions enables readers to gradually update their mental representation of the text resulting in a coherent situation model (van der Schoot et al., 2008). Notably, the ability to construct and update situation models is considered not only at the heart of reading comprehension. Rather, it is seen as the core component of comprehension in general, including comprehension of oral language. For example, it is in this context that we can understand the simple view of reading introduced by Gough and Tunmer (1986), which states that reading comprehension ability can be decomposed into word decoding and listening comprehension.

The most distinctive aspect of situation model construction is the need for coherence (McNamara and Magliano, 2009; van den Broek et al., 2011). Texts usually do not describe narrative situations completely and in full cohesion. Rather, they often pose comprehension problems due to implicitness of information, semantic vagueness and ambiguities, or temporal, spatial, and causal discontinuities (e.g., Zwaan et al., 1995). When a particular text segment lacks information required to obtain sufficient coherence, readers need to supplement their situation model representation with inferences by drawing upon prior knowledge or text clues (see below; Cain and Oakhill, 2007; van den Broek and Espin, 2012). Only then, they can infer what is implied, resolve vagueness and ambiguities, or fill gaps in causal, spatial and temporal descriptions. In this regard, situation models constitute the level of text representation which is associated with deep processing (van der Schoot et al., 2010). In contrast to surface-level and textbase representations, situation models are not restricted to only the actual textual information; rather, they are “amalgamations from information stated explicitly in the text and inferences” (Zwaan and Radvansky, 1998, p. 163). Generally, the relationship between situation model construction and inference generation is considered to be a mutual one, that is, inferences are critical for constructing situation models, and situation models facilitate inference generation (e.g., Rickheit et al., 1985; Graesser et al., 2001). Here, we focused on the first direction of influence, that from inference generation to situation model construction. Or, to put it in terms of the training: our training program was designed to enhance children’s inference making skills contributing to situation model construction and hence deep text comprehension.

### The Inference Making Training

The inference making training was structured in a sequence of alternating instruction lessons (conducted in groups) and computer-based lessons (conducted individually). To teach children why inference making skills are important and how they can be applied, the instruction lessons made a major distinction between what in the literature on inference generation during text comprehension is referred to as knowledge-based and text-based inferences (e.g., Graesser and Kreuz, 1993). That is, children were taught how to make inferences using their prior knowledge (i.e., knowledge-based inferences) or information presented elsewhere in the text (i.e., text-based inferences). In particular, knowledge-based inferences have been proposed to contribute to the situation model level of text representation (e.g., McNamara and Magliano, 2009). In support of this, Radvansky et al. (2001) even pointed out that the “creation of a situation model is essentially an inference-making process in which the given information and general world knowledge is used to construct an understanding of the described situation” (p. 156). Or, to put it in terms of the construction-integration model (Kintsch, 1988): readers draw knowledge-based inferences from text to build a situation model by integrating their existing world knowledge with the information in the propositional textbase. In our training program, children were encouraged to generate knowledge-based inferences by presenting them with passages of text wherein the basic messages could not be easily understood without making knowledge-based inferences.

In addition to knowledge-based inferences, integrated situation models are also constructed from inferences readers make on the basis of textual clues (e.g., McNamara and Magliano, 2009). It is generally agreed upon that in reading comprehension, readers need to connect incoming information to previous information to construct a coherent situation model from the text (e.g., Zwaan et al., 1995). Since texts almost never contain fully explicit descriptions, this mapping process requires readers to make text-based inferences in order to understand what is implied and fill in the gaps left by the author. By making connections between text constituents, they can infer the implicit relationships among the propositions in a text and hence (re-)establish coherence in their situation model (e.g., Kintsch and Rawson, 2005; Perfetti et al., 2005).

Although the texts used for our training required readers to make different kinds of inferences,– for example, inferences concerning the setting of a story, or inferences about the internal states of characters including their intentions and emotions – they were particularly designed to promote the generation of causal inferences. Causal inferences are considered critical for obtaining coherence in situation models because they explain why things happen in a given context (e.g., Graesser et al., 1994; Langston and Trabasso, 1999). As such, causal inferences help readers to link the bits and pieces of text into a coherent whole. For example, look at the following sentences: “The campfire started to burn uncontrollably. The boy scout grabbed a bucket of water” (example adopted from Bowyer-Crane and Snowling, 2005). To make sense of the latter sentence, readers should relate it to the former and have to infer that the boy scout mad an attempt to extinguish the fire. For this, they should activate the mediating idea that ‘water extinguishes fire’ from their prior knowledge available in long-term memory (Singer et al., 1997; Bowyer-Crane and Snowling, 2005).

In the instruction lessons on both text-based and knowledge-based inferencing strategies, it was explained to children that generating inferences helps them (1) to resolve coherence breaks (such as vagueness of meaning, ambiguities, and discontinuities) encountered when reading particular passages, and (2) to understand what is, or could be, implied in a text but not explicitly stated. Although both purposes of inference generation largely overlap, they differ in the extent to which implicitness of information leads to actual problems of understanding and interpretation disturbing the ‘flow’ of comprehension (e.g., Beeman et al., 2000). The more it does (purpose 1), the more readers will feel externally motivated, by the text, to generate an inference and restore coherence. The less it does (purpose 2), the more their decision to generate an inference has to come from internal motivation to go beyond the propositional content of the text, and embellish on the story being described. The above distinction comes close to the difference between bridging and elaborative inferences made in the inference making literature (e.g., Millis and Graesser, 1994). Bridging inferences (or coherence-based inferences as they are also called) are required in order to (re-)establish coherence within a text, and can be generated by referentially tying a word or a clause that has just been read to a previously read word or clause (e.g., Schmalhofer et al., 2002). In contrast, (predictive) elaborative inferences are not required to comprehend text, but they help in enriching reader’s mental text representation based on their related world knowledge and personal experiences (e.g., Bowyer-Crane and Snowling, 2005). In our inference making training, we took the opportunity to address this difference in an integrative manner when explaining the children to resolve coherence breaks and to infer the information implied in a text.

In teaching text-based inferences, emphasis was placed on two aspects of inference generation: making single lexical inferences and linking multiple lexical inferences together to make sense of what is really happening in a story (see also Yuill and Oakhill, 1988). With regard to the former, children were encouraged to look for important word clues and to reflect on, and utilize, the information they provided about the meaning of the text. For example, texts contained keywords (e.g., ‘menu,’ ‘towel,’ and ‘sand’) from which the setting of a story could be inferred (restaurant, bathroom, and beach, respectively). Subsequently, they were taught how to combine clues in order to acquire a more complete and refined picture of what was most probably going on in the story. Continuing on the importance of the setting of a story, it was explained to children that in the light of the setting of a story, vague or ambiguous information presented elsewhere in the text can be resolved. Consider, for example, the sentences ‘Peter put the candle-lit cake on the table. He wondered what present his little sister would get.’ By using the story setting inferred from the words ‘candle-lit cake,’ readers might deduce that Peter’s sister was about to receive presents for her birthday, not for Christmas or some other occasion. Over the course of the inference making training, children were specifically challenged to chain inferences together in order to create a coherent causal situation model. In doing so, it was emphasized that backward as well as forward clues in the text can be used to understand what is implied and (re-)establish coherence between sentences.

In the instruction lessons, children were provided with a wealth of appealing text examples to illustrate the different explanations. In both the instruction and computer-based lessons they were required to make exercises which were specifically designed to induce the inferential processes taught to them. The exercises included question-answering and CLOZE techniques both of which were used in a progression from simpler two-sentence texts (beginner texts) to more difficult, longer multi-sentence texts (advanced texts).

### Evaluating the Training’s Effectiveness

As mentioned above, the inference making training was developed to improve children’s inference making skills contributing to situation model construction and therefore deeper text comprehension. To evaluate the effectiveness of the training, we used the probe verification task (e.g., McDaniel et al., 2001; Yang et al., 2007; Friese et al., 2008) to measure level of mental text representation at pre- and post-test. In this task, participants read short text scenarios. After each scenario, a probe statement was presented. Children had to decide whether or not this statement was a good title for the scenario. Probe statements differed with respect to the level of text representation (surface, textbase, situation model) required to make the yes/no decision. Since the inference making training was specifically targeted at enhancing the key skill in situation model construction (i.e., inference making), we hypothesized that our training would lead to a significant improvement of post-test relative to pretest performance on the probe verification task. More in particular, we expected that, after the training, children in the experimental group would show longer response times (indicating greater effort involved in creating coherent meaning from text; e.g., Rayner and Pollatsek, 1989) and higher accuracy rates in the (inference) condition in which they were required to base their yes-or-no decisions on a situation model representation of the presented scenarios.

Additionally, we anticipated the inference making training to result in higher levels of general reading comprehension. This was motivated by the idea that inference making is essential to (teaching) reading comprehension and, as such, we hypothesized that benefits arising from the training would transfer to other texts than those developed with the specific experimental purposes (i.e., the probe verification task) in mind. To raise the probability that improved inference making skills, as a result of the inference making training, would transfer to general reading comprehension, we led children practice with narratives that differed in difficulty, length, and type of implicit information (also see Wassenburg et al., 2015). Additionally, in an attempt to foster transfer of the learnt inference making skills, naturalistic texts adapted to children’s own personal experiences were used.

Finally, we also explored to what extent the inference making training influences reading motivation. Particularly, children’s attitude toward reading comprehension may become more positive because the training was specifically developed to help children gain a deeper understanding of text. In doing so, we departed from the assumption that by teaching children how to make knowledge-based and text-based inferences, they will be better equipped to move beyond understanding a text at the propositional level, and form a non-linguistic, situation-based representation of what it is about. In particular, the latter type of inferencing might lead to increased motivation to read. From research on the relation between reading (comprehension) and motivation, we know that children who connect information in a text to their own background knowledge and experiences, build a richer and more vivid mental representation from the text, which in turn, leads to increased motivation to read more (e.g., Van Sluys, 2008; Taboada et al., 2009; Retelsdorf et al., 2011). Noticeably, such an improvement of reading motivation would be specifically desirable because of its bidirectional relation with reading comprehension, which was previously shown by numerous studies involving primary school children (for an overview see Morgan and Fuchs, 2007).

## Materials and Methods

Please note that parts of the descriptions of the sample, trainings, and procedures are taken from an article by Wassenburg et al. (2015), which, as already mentioned in Section “Introduction,” covers another aspect within the same overarching intervention study conducted by our research group.

### Participants

Participants were 143 third (age range: 8 years and 3 months – 9 years and 11 months) and fourth (age range: 8 years and 10 months – 12 years) graders from six regular, average performing primary schools in a large urban area in the Netherlands. The schools where the intervention took place had a collaboration with the university, but participation in the intervention study was voluntary. In accordance with a procedure preferred by the schools and endorsed by the ethical committee of the faculty, parents were provided a letter about the aim and methods of the study. They could allow or deny the participation of their child by returning a preprinted objection note.

Children with dyslexia and/or an IQ less than 85, as indicated by school records, were excluded. In addition, we excluded children for which school records indicated (diagnosed) problems, pointing to developmental or intellectual disadvantages. This resulted in removal of 25 children from the initial sample. Of the remaining children, 67 children participated in the inference making training group and 51 children formed the control training group, which followed the school’s regular reading comprehension curriculum.

Random assignment of children was not possible due to practical and organizational reasons imposed by the schools. For example, schools preferred not to make within-class divisions between groups of experimental and control children.

However, after carefully assigning classes to conditions, it turned out that the two groups were comparable on age, socio-economic status^1^, gender ratio, class size, decoding [indicated by raw scores on a standardized Dutch word reading test (Een Minuut Test; Brus and Voeten, 1999)] and IQ (indicated by raw scores on Raven’s Standard Progressive Matrices – Short Form). Group characteristics are presented in **Table 1**.

**Table 1:** Characteristics for each group.

	Control group	Training group	*t* (df)
	Mean (*SD*)	Mean (*SD*)	
Age (years:months)	9:8 (0:9)	9:8 (0:8)	-0.13 (115)
Socio-economic status	0.54 (0.62)	0.57 (0.54)	-0.27 (116)
Gender ratio (% of boys in class)	50.07 (7.11)	50.25 (6.56)	-0.14 (116)
Class size	26.27 (4.72)	25.43 (4.14)	1.03 (116)
Decoding	67.90 (13.42)	63.72 (15.85)	1.51 (115)
IQ	21.38 (3.93)	21.28 (4.00)	0.14 (113)

*Socio-economic status was inferred from area of residence, Decoding = raw scores on a standardized Dutch word reading test (Een Minuut Test; Brus and Voeten, 1999), IQscore = raw scores on Raven’s Standard Progressive Matrices – Short Form. All statistical comparisons were non-significant (p > 0.14).*

### Design

The study used a pretest–posttest control group design wherein training group (inference making vs. control) was the independent variable and situation model-based inference making ability (i.e., the reading skills which were trained), level of general reading comprehension, and reading motivation were the dependent variables. Pretests and post-tests were administered individually by trained research assistants in the 2 weeks before and after the inference making and control training, and consisted of different versions of the same tests. We counterbalanced the order of the tests across participants.

### The Inference Making Training

Inference making was taught in a 4-weeks training program containing eight 30-min lessons (two lessons per week). Specifically, half of the lessons were instruction lessons conducted in groups (lessons 1, 3, 5, and 7), the other half were computer-based lessons conducted individually (lessons 2, 4, 6, and 8). Instruction lessons and computer-based lessons were taught alternately, so each instruction lesson was followed by a computer-based lesson. All lessons consisted of a balanced approach of direct instruction, modeling, guided practice and individual practice (Houtveen and van de Grift, 2007). Particularly, depending on the type of lesson, relatively more time was spent on direct instruction, modeling, and guided practice (in the instruction lessons), or on guided and individual practice (in the computer-based lessons). Lessons were conducted by trained research assistants. They followed standardized instructions and had received approximately16 h of training before the start of the training phase.

To promote the children’s engagement and motivation for the training, we took into account the following aspects. First, the training program was presented to the children as a ‘detective training’ meant to teach them, among other things, how to ‘hunt for’ clues in a text or their background knowledge in order to gain a more complete and in-depth understanding of what they are reading about. Second, an abundant variety of relevant, level-appropriate, and appealing text examples was used to explain the different instructions, and guide the children in reaching the training goals. Third, we used scaffolding techniques including (i) gradual fading of teacher support and (ii) transfer of responsibility from the teacher to the students once the latter were beginning to become more competent (Guthrie et al., 2007; Houtveen and van de Grift, 2007). Finally, each lesson ended with a reflective discussion about what and how the children had learned and why that is important.

#### Instruction Lessons

Instruction lessons were provided to children in groups of 5–6 in a separate classroom. Instead of relying primarily on a top–bottom approach, the instruction lessons were collaborative and interactive, with students in the role of engaged learners. The goals of the training were to teach children why inference making skills are important and how they can be applied. The inference making skills which were taught and practiced could be used for knowledge-based or text-based inferences; that is, children had to make inferences using their prior knowledge or information presented elsewhere in the text. Children were explained that skilled adult readers make these different types of inferences in order to (i) understand what is, or could be, implied in a text but not explicitly stated or (ii) to resolve coherence breaks (i.e., comprehension difficulties including vagueness of meaning, ambiguities, and discontinuities) encountered when reading particular passages.

To illustrate this, children had to, among other things, select keywords in texts, and indicate what information they gave about the text (i.e., make lexical inferences). As in the inference training by Yuill and Oakhill (1988), children were stimulated to combine single lexical inferences. For instance, by using the story setting inferred from the word ‘wave,’ readers might deduce that the word ‘tower’ referred to a sandcastle (example taken from Yuill and Oakhill, 1988). In explaining the importance of inferences for maintaining coherence in the reader’s mental representation of a text, it was emphasized that backward as well as forward clues in the text can be used to understand what is implied, resolve vagueness, ambiguities or discontinuities, and repair comprehension. Within lessons, texts increased in length, from single sentences or two-sentence texts to multi-sentence discourses. At the end of each instruction lesson, children practiced, with paper and pencil, with the type of exercise they had to make in the subsequent computer-based lesson (see below), namely question-answering/beginner exercises in lesson 1, CLOZE/beginner exercises in lesson 3, question-answering/advanced exercised in lesson 5, and CLOZE/advanced exercises in lesson 7.

From the first lesson onward, children were provided with a set of six practical guidelines. These guidelines coincided with the goals set out for the training and served as a means to help the children perform, and learn from, the different exercises used to induce inferential processes. Also, throughout the training, the group-based discussions about how to carry out the inferential processes centered around these guidelines. Inevitably, the different inference making skills which were trained were related and difficult to isolate. As a consequence, the presented guidelines overlapped in their content and use, and they were taught in an integrative manner in all lessons. Besides their prominent, recurrent role in the instruction lessons, the guidelines were incorporated in the exercises children had to practice in the instruction lessons and perform in the computer-based lessons (see below). Exercises were presented interchangeably and with progressive difficulty. In **Table 2**, the overall structure of the training program as well as two text examples of each guideline are presented.

**Table 2:** Overview of the overall structure of the training program including two text examples of each guideline.

		Guideline	Example 1	Example 2
Text-based inferencing	Use clues to understand what is, or could be, implied	Look for clues	(1) Peter wondered what present his little sister would get.	Little Tim brought me rifle to his shoulder and confidently hit the target.
		Combine Clues	(2) Peter put the candle-lit cake on the table. He wondered what present his little sister would get.	Little Tim won a huge stuffed animal. He brought the rifle to his shoulder and confidently hit the target.
	Use clues to resolve vagueness, ambiguities, or discontinuities	Backward searching	(3) The sand tickled Trudy’s toes. [...←...] Her castle made Trudy proud.	This was not Chris’ favorite lesson, he had always been better with words. [...←...] He looked at the tables one more time.
		Forward searching	Curiously, Mary looked at her new face in the mirror. [...→...] (4) Luckily, the bruises faded and she was allowed to leave the hospital soon.	Lisa almost stepped on the town hall.[...→...] Like most children, she was excited about the miniature park and how they reconstructed the city in all its details.
Knowledge-based inferencing	Use prior knowledge to understand what is, or could be, implied		(5) It was a warm summer right in the park. Anne and David really enjoyed what they saw. Anne bought a cinnamon stick and David purchased candy floss. Lights were flickering everywhere. Anne was very excited. She took a ride on a small train. They walked home after they had spent all their money.	Finally there was enough wind, which made David and his father decide to head for the beach. “I really hope it will work.” David said. They had spent at least 2 h of tinkering, in the shed next to their house. At first they made one out of a plastic bag, but this one did not even withstand the test flight in their backyard.
	Use prior knowledge to resolve vagueness, ambiguities, or discontinuities		(6) Lisa and Susan played in shallow water. The girls were throwing around a large inflatable ball between them. While attempting to catch the ball Lisa suddenly stepped in a piece of glass from a broken bottle. For the remainder of the afternoon. Susan reluctantly joined Lisa in reading magazines while lying on their beach towels.	The lame old man still tried to wrap his head around the news his doctor just shared with him when he stepped outside. Walking by a toy store, he suddenly realized that he had to rush as he would otherwise arrive too late at his grandson’s birthday party. He accelerated his pace just before entering the busy intersection without even noticing the roaring engines passing by. Blaring sirens sounded when the ambulance set off to the hospital.

#### Computer-Based Lessons

In the computer-based lessons, the purpose was to (1) offer children the opportunity to engage in additional practice with the inference making skills learned in the instruction lessons, (2) practice these skills individually, and (3) doing so in a way that aligns with current educational practice where the computer is being increasingly used during reading comprehension lessons. The computer-based lessons, which took place in a separate (computer) classroom, contained question-answering (lessons 2 and 6) and CLOZE techniques (lessons 4 and 8) to trigger the inferential processes which were taught in the instruction lessons. Both techniques were used in a progression from two-sentence texts (beginner texts used in lessons 2 and 4) to longer multi-sentence (8 ± 3) texts (advanced texts used in lessons 6 and 8). In the question-answering procedure children had to read texts, after which they were required to answer inference questions concerning, for example, causal antecedents and consequences, or character intents and emotions. The CLOZE tests required children to fill in blank spaces in a text. To complete the blanks, and bring closure to the text, children needed to understand a sentence in relation to the text that contained it.

As explained above, to generate correct inferences in the computer-based lessons, children had to employ both backward and forward searching strategies to locate the appropriate clues in the text (text-based inferencing) and/or they had to integrate the text information with their existing world knowledge (knowledge-based inferencing). In all computer-based lessons, after giving the answer, children had to indicate the words which helped them to make the required inference.

Although the assignments during the computer-based lessons had to be made individually, the beginning and end of these lessons were held in groups. With the children being assigned to small groups of 5–6 children, each computer-based lesson started with a recapitulation of what was taught in the preceding instruction lesson and ended with a discussion in which the children reflected on the what, how and why of the strategic reading activities which were trained.

### Training for the Control Group

Children in the control group followed the regular curriculum taught by their own teachers. This meant that they attended reading comprehension lessons twice a week, just like the children in the experimental training group. For the reading comprehension lessons one of the most popular reading comprehension methods in the Netherlands (i.e., Nieuwsbegrip) was used. These lessons involved whole class reading as well as small group reading instruction at children’s appropriate reading level combined with individual practice. Reading strategies which were taught included predicting (i.e., using text characteristics such as the title and headings before reading the text to make predictions about what the text will be about, which involves thinking ahead and anticipating information and events in the text.), clarifying (i.e., identifying unfamiliar or difficult words and phrases and learning how to get to the appropriate meaning or interpretation), and summarizing [i.e., learning to extract the key information described in (part of) a text in order to get a more concise understanding of the main ideas and consolidate important details related to it].

### Pre- and Post-tests

Pre- and post-tests were administered at school. Children individually completed the probe verification task in a silent room, and the reading comprehension test and the reading motivation questions were completed in the classroom using a whole-class test taking approach.

#### Probe Verification Task

In the pre- and post-tests, inference making abilities were measured using a probe verification task adapted and translated to Dutch from, among others, Friese et al. (2008). The task consisted of 84 two-sentence text scenarios presented on the computer screen. The scenarios described stereotypical situations such as ordering at McDonald’s or stepping on broken glass while barefoot. After each scenario, a two-word probe statement was presented which represented the typical outcome of the situation (e.g., eating hamburgers, cut foot). Children had to decide whether or not this statement was a good title for the scenario by pressing a ‘yes’ or ‘no’ key on the keyboard.

By modifying the ending of the scenarios, different relations were established between the probe statements and scenarios. There were four experimental conditions, each with 14 trials (i.e., scenario/statement combinations). In **Table 3**, a sample scenario is presented in each experimental condition. In the explicit condition, the probe statement could be typified as an explicit repetition of the scenario’s ending. In the paraphrase condition, the scenario ended with a paraphrase of the probe statement. In the inference condition, the scenario did not mention the probe statement but provided enough information to infer it as a plausible outcome of the described situation. In the unrelated condition, the probe statement was consistent with the situation but the scenario did not give any logical explanation for it. In contrast to the other statement types, unrelated statements therefore required a ‘no’ response. As a result of our manipulations, children had to base their yes/no-decision in the inference and unrelated condition on a situation model representation of the presented scenario, but could rely on a surface and textbase representation in the, respectively, explicit and paraphrase condition. In the remainder of this paper, less weight will be given to the unrelated condition, since the interpretation of the results in this condition was complicated due to the fact that “no” responses had to be given for the unrelated statements while for the other conditions we looked at “yes” responses (see also Ratcliff and Hacker, 1981; Ratcliff, 1987; Friese et al., 2008).

**Table 3:** Sample scenario and required response to the probe statement ‘wine spilled’ in each condition in the probe verification task.

Condition	Scenario	Required response
Explicit	The flight attendant served the passenger red wine. At that moment, turbulence caused the wine to *spill*.	Yes
Paraphrase	The flight attendant served the passenger red wine. At that moment, turbulence caused the wine to *splash*.	Yes
Inference	The flight attendant served the passenger red wine. At that moment, turbulence *occurred which was very severe.*	Yes
Unrelated	The flight attendant served the passenger red wine. At that moment, *the plane was at cruising altitude*.	No

*Example taken from Friese et al. (2008).*

Each scenario consisted of 18 (±1) words. The first sentence (seven words) as well as the first three words of the second sentence were identical in all conditions (based on Dutch sentences). The remainder of the second sentence varied across conditions as explained above. To make sure that each scenario appeared equally often in all conditions across participants, we arranged the total set of scenarios in four material sets and counterbalanced sets and conditions by a Latin square (see Friese et al., 2008). To balance the ratio of “yes” and “no” responses, we included 28 filler trials which had to be answered with “no.” In the filler trials, statements had nothing to do with the preceding scenarios. Responses to filler items were excluded from the analyses.

The probe verification task started with five practice trials. When an incorrect answer was given, the test leader explained to the children why the answer they had chosen was wrong. During the experimental trials no further explanations or feedback were given. The task lasted approximately 30 min. The probe verification task had a very good internal consistency given that the Cronbach’s alpha’s were 0.82 in the pretest and 0.91 in the post-test.

#### Reading Comprehension

The Grade 3 and Grade 4 versions of the standardized CITO Reading Comprehension Test were used to measure children’s reading comprehension skills (Institute for Educational Measurement, 2010). This test is part of the standard Dutch pupil monitoring system and is designed to determine general reading comprehension level in primary school children. It contains two modules, each consisting of a text and 25 multiple choice questions. The questions pertain to the word, sentence or text level and tap both the text base and situation model representation which readers can construct from texts (Kintsch, 1998). For each student, the total score on all items was converted into a normed proficiency score. The rescaling procedure enabled us to compare the results of the pre- and post-test versions of the CITO Reading Comprehension Test. In addition, the obtained proficiency scores made it possible to compare the scores of children from a different grade (i.e., Grades 3 and 4). The internal consistency coefficient of the tests was high with Cronbach’s alpha’s not less than 0.85 (Feenstra et al., 2010).

#### Reading Motivation

**FIGURE 1:**
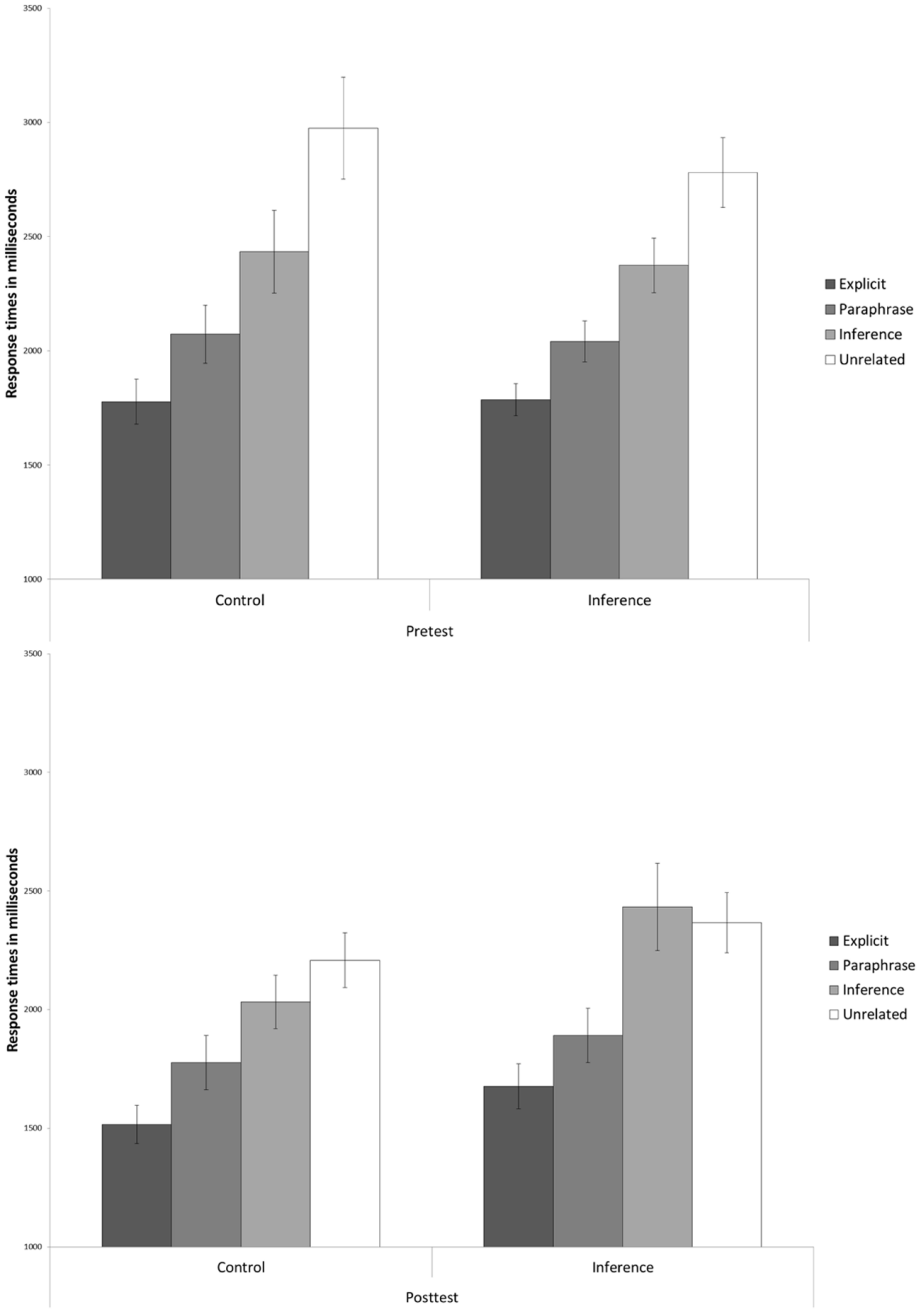
**Response times to the probe statements (in milliseconds) presented as a function of Probe Statement condition (explicit vs. paraphrase vs. inference vs. unrelated), Training Group (inference making vs. control) and Time (pretest vs. post-test; CSE)**.

We asked the children how much they liked reading comprehension before and after the training. Children had to answer on a four-point Likert scale represented by cartoon figures (1 = I do not like it at all; 4 = I like it a lot). After this, children were provided the opportunity to orally explain why they did or did not like reading comprehension. The oral answers were not documented. Their purpose was to give the children the chance to provide their opinion and express their wishes for improvement.

## Results

### Probe Verification Task

#### Response Times

In **Figure 1**, the correct response times to the probe statements (in milliseconds) is presented as a function of Probe Statement (explicit vs. paraphrase vs. inference vs. unrelated), Training Group (inference making vs. control) and Time (pretest vs. post-test). On the response times, a 4 × 2 × 2 × 2 analysis of variance (ANOVA) was performed with Probe Statement and Time as within-subject variables, and Training Group and Grade (Grade 3 vs. Grade 4) as the between-subject variables. Although there was a main effect for Grade, Grade did not interact with any of the other variables (all *F*-values < 2.00, all *p*-values > 0.10). Therefore, results were averaged across Grade in the subsequent analyses. Additionally, main effects for the factors Time and Training Group are not reported given that these results do not contribute to providing an answer to the hypotheses and can only be meaningfully interpreted when these two factors are combined.

As can be seen from **Figure 1**, the probe verification task showed a main effect of Probe Statement [*F*(3,333) = 74.11, *p* < 0.001, $\eta_{p}^{2}$ = 0.40], reflecting the expected increase in response latencies from the explicit condition (in which decisions could be based on the surface representations of a text scenario), via the paraphrase condition (in which decisions required a text base representation), to the inference and unrelated conditions (in which decisions required a situation model representation). As such, we replicated the results of previous studies (e.g., Friese et al., 2008), and reinforced the validity of the probe verification task.

Of more interest are the pre- and post-training group differences in task performance. At pretest, the inference making and control training groups performed similarly in all probe statement conditions [Probe Statement × Training Group: *F*(3,339) = 1.60, *p* = 0.16, $\eta_{p}^{2}$ = 0.02]. However, in the post-test, the performance of the experimental and control training groups was different. In particular, children who had received the inference making training tended to show larger inference–paraphrase probe statement differences in response time [Probe Statement × Training Group: *F*(3,336) = 2.38, *p* = 0.07, $\eta_{p}^{2}$ = 0.02]. The above findings were confirmed by a significant interaction between Probe Statement, Training Group, and Time [*F*(3,333) = 2.62, *p* = 0.05, $\eta_{p}^{2}$ = 0.02]. Planned pairwise comparisons were conducted to further examine this three-way interaction. The only significant group difference which was found was between the paraphrase and inference condition in the post-test [Probe Statement × Training Group: *F*(1,111) = 5.02, *p* = 0.03, $\eta_{p}^{2}$ = 0.04]. In sum, the results indicate that, after the inference making training, children took more time making a yes-or-no decision in the probe verification task when they were required to base their decision on a situation model representation of the scenario (in the inference condition), but not when they could base their decision on a text base representation (in the paraphrase condition) or surface representation (in the explicit condition).

#### Accuracy Rate

**FIGURE 2:**
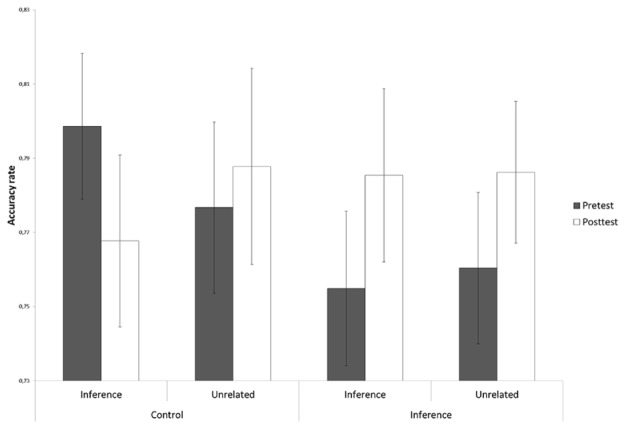
**Accuracy rate in the probe verification task presented as a function of Training Group (inference making vs. control) and Time (pretest vs. post-test) for the inference probe statements and unrelated probe statements (CSE)**.

**FIGURE 3:**
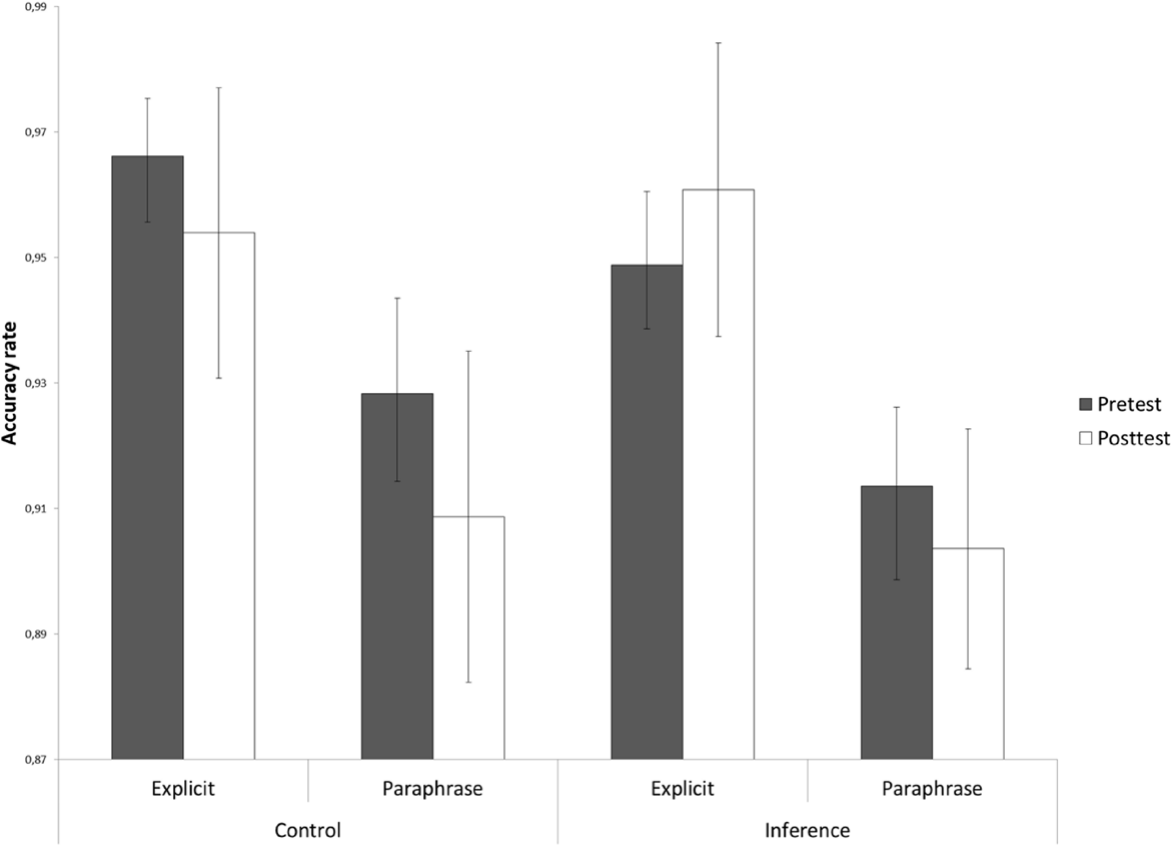
**Accuracy rate in the probe verification task presented as a function of Training Group (inference making vs. control) and Time (pretest vs. post-test) for the explicit probe statements and paraphrase probe statements (CSE)**.

Importantly, the accuracy data showed that, for the inference probes, children profited from the extra effort they invested in making the verification decisions as the increase in response time yielded a higher accuracy rate. This can be clearly seen in **Figure 2**, where the percentage of correct responses to the inference probes is presented as a function of Training Group and Time. A significant improvement of post-test relative to pretest performance was only observed for children in the inference making training and not for children who had followed the control training [Training Group × Time: *F*(1,111) = 3.87, *p* = 0.05, $\eta_{p}^{2}$ = 0.03]. For the unrelated probes (**Figure 2**), on the other hand, neither the inference making training nor the control training led to a significant increase in accuracy rate [Training Group × Time: *F*(1,111) = 0.02, *p* = 0.90, $\eta_{p}^{2}$ = 0.00]. As hypothesized and in line with the response time results, no Training Group by Time interactions for accuracy rate were obtained for the explicit and paraphrase probe statements (*p*’s > 0.74, see **Figure 3**).

### Reading Comprehension Performance and Reading Motivation

**FIGURE 4:**
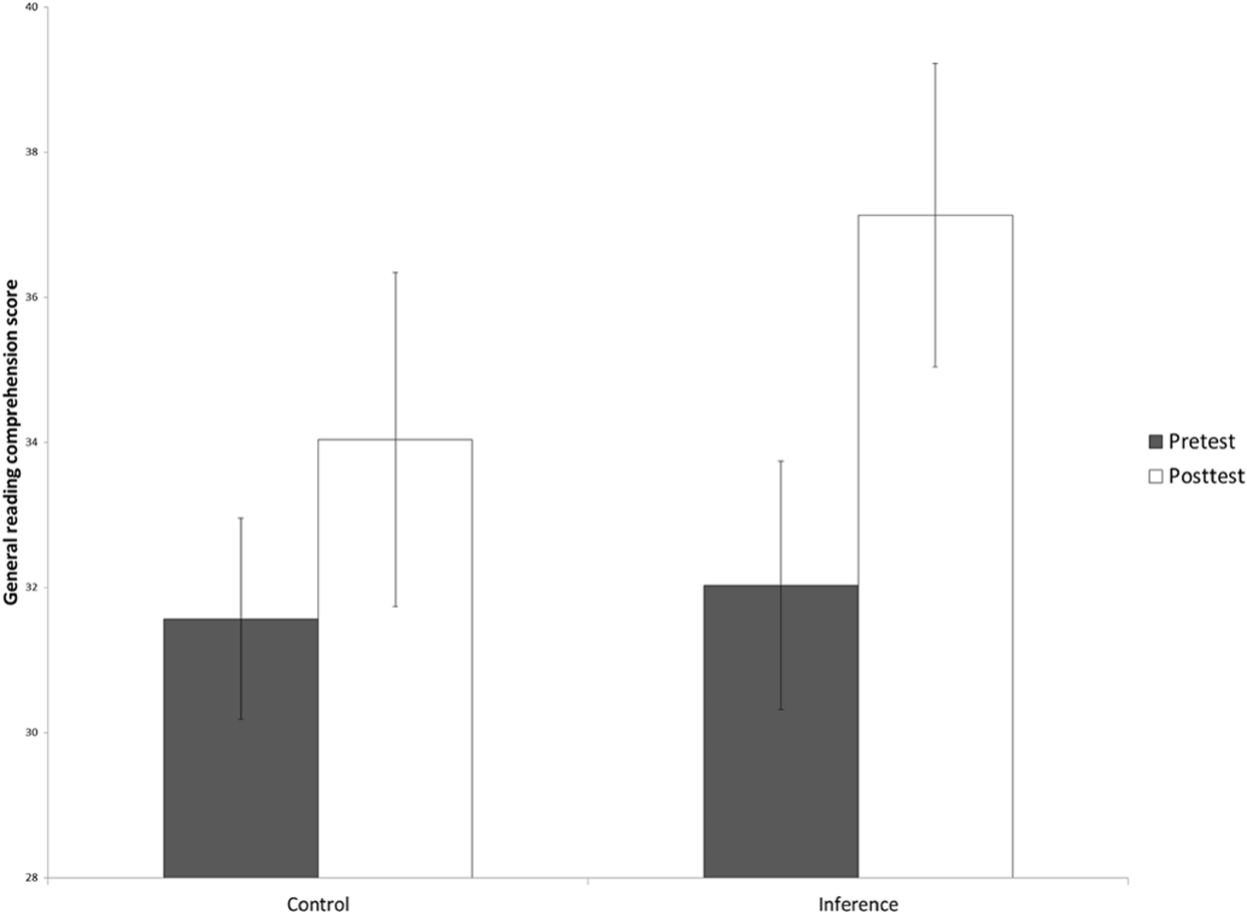
**Proficiency scores on the standardized, normed CITO Reading Comprehension Test presented as a function of Training Group (inference making vs. control) and Time (pretest vs. post-test; CSE)**.

Besides improved performance on the probe verification task, children in the inference making training showed gains in a general measure of reading comprehension. As displayed in **Figure 4**, the inference making training resulted in higher reading comprehension scores on the standardized CITO Reading Comprehension Test. Children in the control group did not show this effect. On the reading comprehension proficiency scores, we conducted a 2 × 2 ANOVA with the within-subject variable Time and the between-subject variable Training Group. The results of the ANOVA analysis showed a significant interaction between Training Group and Time [*F*(1,108) = 4.20, *p* = 0.04, $\eta_{p}^{2}$ = 0.04].

**FIGURE 5:**
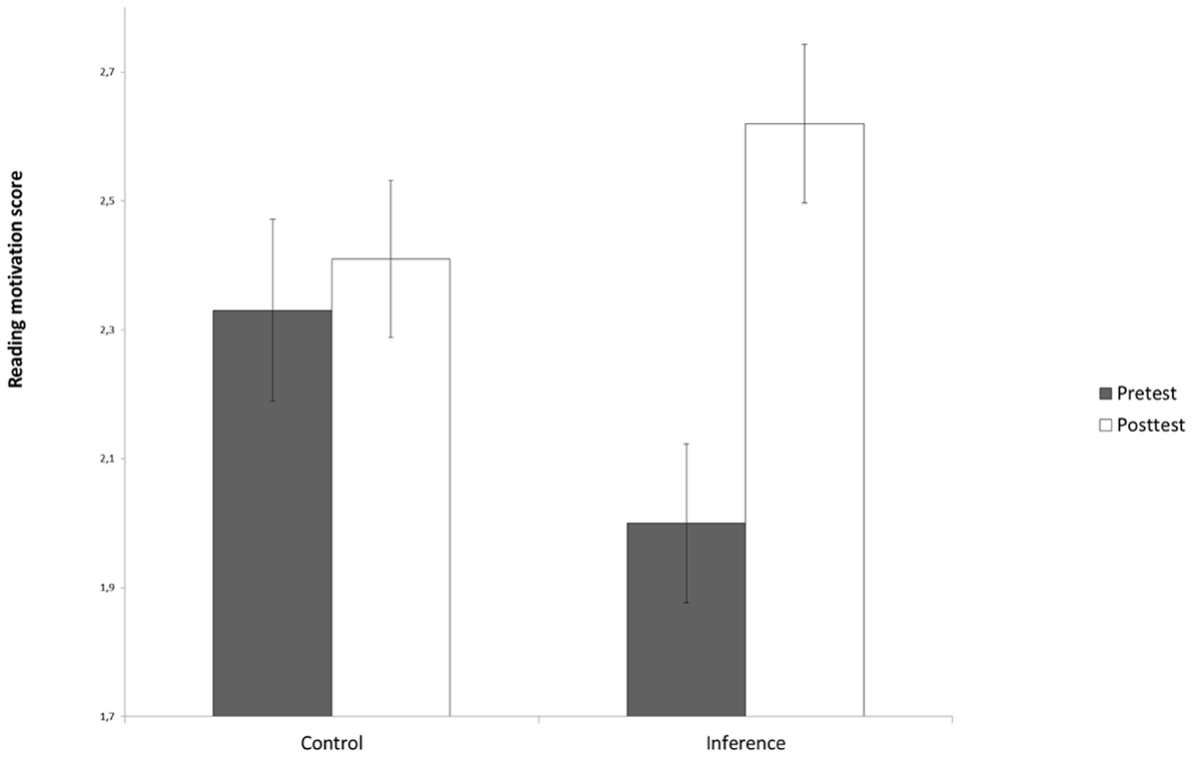
**Reading motivation score presented as a function of Training Group (inference making vs. control) and Time (pretest vs. post-test; CSE)**.

In addition to the gains in general reading comprehension, the inference making training led to higher reading motivation: children indicated that they enjoy reading more after the training than they did before (see **Figure 5**). Again, this effect was not observed in the control group, resulting in a significant Training Group × Time interaction [*F*(1,110) = 7.06, *p* = 0.01, $\eta_{p}^{2}$ = 0.06].

## Discussion

This study aimed to enhance text comprehension at the situation model level in primary school children. Extending beyond literal and propositional representations, situation models are constructed when readers integrate information across the text and information in text with background knowledge into a coherent mental representation of what is happening in a story (e.g., Zwaan and Radvansky, 1998; van den Broek et al., 2011). To this end, we developed a reading strategy training targeted at strengthening children’s inference making skills, which are generally regarded as essential for situation model construction (e.g., Radvansky et al., 2001; McNamara and Magliano, 2009). In doing so, the situation model theory not only served as our framework within which the training program was set up, it was also used to guide the evaluation of its effectiveness. That is, we used the probe verification task (e.g., McDaniel et al., 2001; Yang et al., 2007; Friese et al., 2008) to measure level of mental text representation (surface, textbase, situation model) at pre- and post-test. To the best of our knowledge, we are the first to apply such an all-encompassing ‘level of text representation’-approach to teaching inference making skills to third and fourth graders. In the interpretation of the results, the focus will first be on the inference making skills supportive to situation model construction (i.e., the skills in which the children were trained). Then, the transfer of these skills to general reading comprehension will be discussed. Finally, we will take an exploratory look at the motivational effects of our training and draw some overall conclusions.

The first main finding of this study is that the inference making training led to a significant improvement of post-test relative to pretest performance on the probe verification task. After the training, children showed longer verification times in the condition in which they were required to base their yes-or-no decision on a situation model representation of the presented text scenarios (inference condition) compared to the conditions in which they could rely on a textbase or surface representation (respectively, the paraphrase and explicit condition). Children in the control group did not show this effect. This finding can be explained by assuming that the experimental training has caused children to put more effort in generating inferences contributing to a situation model representation of the text they are reading. Importantly, this was not only apparent in the children’s probe verification latencies but also in the accuracy of their responses. Their higher accuracy rate for the inference probes suggests that, due to the additional time they invested in making an appropriate decision, children derived more coherent, situation-based meaning from the text scenarios. It should be added here that, in absolute terms, the pretest-to-posttest gain in accuracy displayed by the inference making training group was small.

However, to fully appreciate this effect, it should be realized that the significant group by time interaction on the probe verification accuracy scores were affected by the control group’s poorer post-test performance relative to their pretest performance. Most likely, this pretest to post-test decline (*p* = 0.04) results from the fact that the probe verification task was somewhat harder in the post-test than in the pretest version. This would undoubtedly provide another, more positive, perspective on the relatively small increase in task accuracy for children in the inference making training. That is, the experimental training group was able to improve their probe verification task performance at post-test despite an increase in task difficulty.

Our second finding is that the inference making training resulted showed a transfer to a general measure of reading comprehension. The children in the experimental training significantly improved from the pretest to the post-test on the used standardized reading comprehension test. There was not such an enhancement observed in the control group in which children received the reading comprehension lessons offered by their school. To understand this finding, it should be noted that our standardized test for general reading comprehension was designed to reflect the textbase and situation model components of the mental representations children construct from narrative texts. Given what was actually taught to the children, the results indicate that especially the increased ability to draw inferences at the situation model level may have been due to our training program. This finding is consistent with the notion that higher-order reading strategies like inference making that support readers to construct a coherent situation model underlies deep-level comprehension of text and that training these skills promotes text comprehension more generally (van der Schoot et al., 2010). We believe that the measures we have taken in developing the text materials used in the training have contributed to the successful transfer of learning. In particular, as mentioned before, children practiced with texts of increasing length and difficulty. Additionally, to further promote transfer of the learnt reading comprehension skills, naturalistic texts were used which were matched to the children’s abilities and interests.

A third issue that we investigated was the extent to which the inference making training resulted in increased reading motivation. Evidently, it is desirable to improve children’s reading motivation given that it positively influences the effort children put into reading (Morgan and Fuchs, 2007). The results of the inference making training on reading motivation are promising in that after this training the children indicated that they liked reading comprehension more, while this was not the case for children in the control training. This result is consistent with at least two pieces of empirical evidence: (1) children who have learned how to connect information in a text to their own background knowledge and personal experiences, which was one of the main goals of our training, build full and more vivid mental representation from text; (2) being able to build such rich mental text representations positively affects children’s level of motivation to read in general (e.g., Van Sluys, 2008; Taboada et al., 2009; Retelsdorf et al., 2011). However, it is important to stress that our study just aimed to offer the first exploratory insights into the extent to which teaching a reading comprehension strategy directed at situation model construction would increase reading motivation. It is for instance not clear from this study whether the one-item Likert scale that we have used is a sufficiently reliable and valid reading motivation measure. Also, this scale did not enable us to get more detailed information on the various aspects involved in reading motivation (Wigfield and Guthrie, 1997). So, a more elaborate questionnaire should be used in future research to investigate more thoroughly what effects reading strategy trainings like the one investigated in this study have on reading motivation.

### Added Values of the Inference Making Training

In our view, the positive effects of the inference making training as described above can be attributed to both the nature of its content and the educational needs of the target group. To start with the latter, the training program, including the materials for training, practice and testing, was specifically tailored to our target group of third and fourth graders. In the Netherlands, children receive formal instruction in reading comprehension for the first time in the second grade. The first year of reading comprehension strategy instruction focuses on basic strategies like writing a summary of the text, making predictions based on text information, and extracting the main message from a paragraph (Palincsar and Brown, 1984; Stoeldraijer and Vernooij, 2007). This lays the foundation for learning the higher-order skills, such as situation model-based inference making, which are essential for deep-level understanding of text. Mastery of the high-level text representation skills should be accomplished later in the primary school’s curriculum, starting at the third grade (Aarnoutse and Verhoeven, 2003). This was taken into account when designing the training program and our findings suggest that by doing so it is possible to develop an effective reading strategy training that is grounded in contemporary literature on inference making in particular and reading comprehension more generally. We are confident that this is not restricted to the strategy trained in the present study (i.e., inference making), as we recently have shown a similar transfer effect to general reading comprehension as a result of training comprehension monitoring skills (Wassenburg et al., 2015).

In addition to its appropriateness for the target group, we believe that also the structure, instructional design, and content of the training program were key factors in its effectiveness. It should be recalled that: (1) the inference making training was structured in a sequence of alternating instruction lessons (conducted in groups and with a focus on direct instruction, modeling, and guided practice) and computer-based lessons (conducted individually and with a focus on guided and individual practice), that (2) all lessons centered around a set of carefully crafted guidelines which were taught in an integrative manner and practiced with increasing difficulty, and, most importantly, that (3) the guidelines were derived from the literature on situation model-based inference making (e.g., Graesser et al., 1994; Kintsch and Rawson, 2005), making distinctions based on the source of an inference (text-based versus knowledge-based), the type of an inference [necessary versus not necessary for (re-)establishing coherence], the depth of an inference (making single lexical inferences versus combining multiple lexical inferences), and the type of searching strategy (forward versus backward). Our study does not allow us to single out the individual contributions for each of these aspects. Even if we would have wished to aim for that, it would not have been very informative as the different kinds of inferences will always show some overlap and are often taught in an integrative manner at school. Rather our study indicates that, together, this set of factors and the carefully crafted and appealing texts have contributed to the effectiveness of the training program we designed (and evaluated) to support inference making skills.

However, we also have to point the reader to some limitations of the study. First, the present results, while significant, are relatively small. Clearly, this reinforces the need to examine the present intervention under realistic, yet carefully controlled, conditions. For example, in a cluster randomized controlled trial (e.g., randomized classroom trial), it should be investigated which teacher-, class-, and school-level variables may influence the course and effectiveness of the inference making training when implemented in actual daily classroom practice. Second, teachers were not involved in the actual training phase. Rather, trained research assistants conducted the training using a standardized protocol to minimize the effects of variables other than the independent variables of interest. It is therefore unknown how the inference making training will work out when it is conducted by teachers in a more naturalistic way. This issue is related to the previous one and should also be investigated in future research. Third, the strategy training has adopted a one-size-fits-all approach, meaning that all children irrespective of their cognitive abilities received the same training in the same way. That is, the training did not take into account the fact that for some children the to-be-learned skills might already be better developed. It is yet unknown whether and how the reported strategy training can be adjusted in a way to adequately meet the individual demands of children who vary in their level of reading comprehension abilities. Again, future research should explore this in more detail. Fourth, it could be argued that, despite our attempt to keep the experimental and control training groups as comparable as possible, children in the experimental training group may have been at an advantage in comparison to children in the control training group. That is, it is impossible to rule out that in the experimental training group factors such as motivation on the side of the learner (e.g., children were told that they participated in a ‘detective training’ whereas children in the control group followed their regular reading comprehension lessons) and/or on the side of the trainers (e.g., the experimental group was trained by trainers that were motivated and enthusiastic about the training) might have played a role in improving performance. Future research should take this aspect into account.

## Conclusion

This study shows that a ‘level of text representation’-approach can provide a useful framework to teach inference making skills to third and fourth graders. Importantly, the only difference between the inference making training and the control training was the content of the trainings (i.e., the number of lessons, its form, and small-group approach were similar between the groups). So, it is unlikely that our findings are the result of other training aspects or alternative explanations, such as instruction time and natural development, than the elements in our inference making training (Houtveen and van de Grift, 2007). Therefore, we conclude that teaching children to create coherent meaning from text through explicit instruction in inference making strategies during only a 4-weeks period effectively enhances situation model construction and hence deep comprehension of text.

## Author Contributions

LB: project manager data collection, development experiments, development intervention, training intervention, data analysis, writing of the article. BK: development experiments, development intervention, data analysis, writing of the article. SW: data collection, development experiments, development intervention. MS: development experiments, development intervention, reviewing article in different stages.

## Conflict of Interest Statement

The authors declare that the research was conducted in the absence of any commercial or financial relationships that could be construed as a potential conflict of interest.

## Footnotes

1 As an indicator of socio-economic status, we used Dutch neighborhood level status scores derived from the national Institute for Social and Cultural Research in the Netherlands. A status score is indicative of a neighborhood’s relative social status in comparison with other neighborhoods. The status score is a compound measure that is computed based on the following features of people living in that neighborhood: average neighborhood income, the percentage of people in that neighborhood who have low incomes, who have a low educational level, and who are unemployed. The 2010 status scores, from which we derived the status scores used in this study, range from -7.19 to 3.19. The higher the status score of a neighborhood, the higher the socio-economic status in that neighborhood (Knol et al., 2012).
